# Integrated multi-omics analysis reveals the response mechanisms of *Trichosanthes kirilowii* to root-knot nematode infection

**DOI:** 10.3389/fpls.2026.1909422

**Published:** 2026-07-28

**Authors:** Lei Zheng, Huadong Wang, Zhiqiang Zhang, Jiuling Gu

**Affiliations:** 1 School of Pharmacy, Sichuan College of Traditional Chinese Medicine, Mianyang, China; 2 Mianyang Science and Technology Bureau, Northwest Sichuan Laboratory of Traditional Chinese Medicine Resources Research and Development Utilization, Mianyang, China; 3 Sichuan Administration of Traditional Chinese Medicine, Mianyang Key Laboratory of Development and Utilization of Chinese Medicine Resources, Mianyang, China

**Keywords:** hormonal changes, multi-omics analysis, root-knot nematode, *Trichosanthes kirilowii*, zeatin biosynthesis

## Abstract

**Background:**

Root-knot nematodes (RKNs) pose a severe threat to *Trichosanthes kirilowii* production, but the molecular mechanisms of its response to RKN infection remain unclear.

**Methods:**

An integrated multi-omics strategy that combined physiological trait analysis, hormone profiling, transcriptome sequencing, and untargeted metabolome analysis was used to systematically clarify the response mechanisms of *T. kirilowii* to *Meloidogyne incognita* infection.

**Results:**

Comprehensive phenotypic observations combined with antioxidant enzyme activity measurements and hormone profiling identified 6 days post-inoculation (dpi) as a critical response timepoint, characterized by initial gall formation, minimum superoxide dismutase (SOD) activity, peak catalase (CAT) activity, and maximal content of auxin (IAA), abscisic acid (ABA), and salicylic acid (SA). Notably, cytokinin-type hormones were significantly upregulated after RKN infection, with zeatin increasing by 202% at 12 dpi and zeatin riboside reaching 38.5-fold that of the control at 24 dpi. Transcriptomic analysis identified 1,705 differentially expressed genes (DEGs), predominantly enriched in plant hormone signal transduction, zeatin biosynthesis, and plant-pathogen interaction pathways. Untargeted metabolomic analysis identified 658 differentially accumulated metabolites (DAMs), primarily involving carboxylic acid derivatives, amino acids, phospholipids, and isopentenyl alcohol esters; combined analysis further revealed that zeatin biosynthesis was the only significantly enriched common pathway; within this pathway, changes in 8 key genes and 5 core metabolites acted synergistically and were significantly correlated with gall number, soluble sugar content, and multiple hormones.

**Conclusions:**

The results indicate that *T. kirilowii* responds to RKN infection through an integrated mechanism involving physiological regulation, hormonal coordination, metabolic reprogramming, and molecular defense, with the zeatin biosynthesis pathway serving as a central hub. These findings provide a basis for molecular breeding and develop green control strategies against RKNs in *T. kirilowii* cultivation.

## 1 Introduction

Root-knot nematodes are among the most destructive plant-parasitic nematodes worldwide and have attracted significant attention due to their extremely broad host range and complex infection strategies (Cazzaniga et al., 2025; Sharma et al., 2025). Global agricultural economic losses caused by root-knot nematodes are estimated to exceed $150 billion annually, with major impacts on vegetable crops (tomato, pepper, and cucumber), legumes, and root/tuber crops across tropical and subtropical regions (Opdensteinen et al., 2024). These losses encompass both direct yield reductions and indirect quality degradation, including altered secondary metabolite profiles in medicinal plants. Following penetration of host root systems by second-stage juveniles (J2), RKNs induce abnormal development of vascular cells into multinucleate giant cells, ultimately establishing permanent feeding sites (Abad et al., 2002). This process triggers dramatic physiological and morphological changes in the host plant, leading to the formation of characteristic galls (Rizvi and Khan, 2023; Sharma et al., 2022). The galls severely impair the host root system’s ability to absorb water and nutrients, leading to stunted growth, reduced yield and quality, and increased susceptibility to other pathogens, thereby raising the risk of mixed infections and exacerbating crop losses (Antil et al., 2023; Kamalnath et al., 2019; Khan et al., 2024). Elucidating the molecular mechanisms underlying host plant responses to RKN infection to accelerate the breeding of resistant cultivars and developing efficient, safe, and sustainable control strategies have become a major challenge requiring urgent breakthroughs in the current field of RKN research.

*Trichosanthes kirilowii* is a perennial climbing herb belonging to the genus Trichosanthes in the Cucurbitaceae family, widely utilized as a traditional medicinal plant across East Asia. Various parts of *T. kirilowii*, including its fruit, seeds, peels, and tuberous roots, are used in traditional medicine and referred to as Gualou (fruit), Gualouzi (seeds), Gualoupi (peel), and Tianhuafen (tuberous roots), respectively (Yang et al., 2019; Yu et al., 2018). These materials exhibit pharmacological activities such as anti-inflammatory, anti-tumor, and immunomodulatory effects and are commonly used for treating chest tightness, constipation, and pulmonary heart disease, demonstrating significant economic and medicinal value (Yu et al., 2018; Zheng et al., 2026, 2025). With increasing demand for *T. kirilowii*, large-scale and intensive cultivation has become prevalent. However, expanded cultivation areas have encountered substantial increases in soil-borne diseases, among which RKN disease has emerged as the primary constraint on production (Jiang et al., 2018). Infection by RKNs not only significantly reduces yields or even causes complete crop failure but also alters the accumulation patterns of secondary metabolites, thereby compromising the quality of the medicinal materials.

The interaction between plants and RKNs involves complex molecular regulatory networks (Singh et al., 2026). During evolution, plants have developed a series of sophisticated defense mechanisms to counter RKN infection, encompassing multiple levels, including physiological regulation, hormone signal transduction, transcriptional reprogramming, and metabolite remodeling (Chen et al., 2024; Dowd et al., 2017; Wang et al., 2023). Advances in multi-omics technologies have provided critical support and novel perspectives for elucidating the molecular mechanisms underlying plant-pathogen interactions. Currently, transcriptome analyses across various plant-pathogen systems have identified numerous differentially expressed genes associated with cell wall modification, reactive oxygen species scavenging, and specialized metabolism; metabolomics offers functional insights into biological processes by characterizing terminal products of cellular activities; integrated analysis combining physiology, hormonomics, and metabolomics facilitates comprehensive understanding of regulatory networks at multiple levels, including physiological responses, hormone dynamics, and metabolic reprogramming, thereby providing a more holistic view of the molecular basis of plant-pathogen interactions (Bali et al., 2018; Dun et al., 2024; Inthaisong et al., 2025; Shahid, 2025). Although significant progress has been made in deciphering the molecular mechanisms of plant-RKN interactions using multi-omics approaches, most studies have focused on model crops such as Arabidopsis and tomato, while research on the medicinal plant *T. kirilowii* remains limited and urgently needs to be strengthened. To address this gap, this study systematically evaluates the multidimensional molecular and physiological characteristics of *T. kirilowii* responses to RKN infection through integrated analysis of physiology, hormonomics, transcriptomics, and metabolomics, aiming to provide a deeper understanding of the regulatory networks and offering a basis for the development of resistant varieties and the optimization of cultivation management.

## 2 Materials and methods

### 2.1 Plant material and RKN inoculation

*T. kirilowii* tissue culture seedlings with uniform growth vigor were selected and transplanted into seedling pots (10 cm × 10 cm × 7.5 cm) containing sterilized substrate, with one plant per pot. After 20 days of growth, the seedlings were inoculated with RKNs (*Meloidogyne incognita*), obtained from Suixian Biological Testing Agricultural Development Company (Zheng et al., 2026). The inoculation procedure involved collecting hatched second-stage juveniles (J2) and preparing a suspension at 1,000 nematodes/mL. Each pot received 1 mL of nematode suspension applied to the rhizosphere soil at a depth of 2–5 cm. A total of 120 pots were inoculated with RKNs, while 30 pots served as controls receiving 1 mL of sterile distilled water (Shukla et al., 2018; Zheng et al., 2026). The seedlings were cultivated under controlled conditions with a temperature of 25 ± 3 °C and a 16-hour light/8-hour dark photoperiod.

### 2.2 Sample collection

Samples were collected at 0 dpi, 3 dpi, 6 dpi, 12 dpi, and 24 dpi. For each time point, three plants were randomly selected and pooled to form one biological replicate, yielding three biological replicates in total. After collection, the samples were rinsed with sterile water and immediately frozen in liquid nitrogen, then stored at -80 °C for physiological, hormonal, transcriptomic, and untargeted metabolomic analyses (Zhang et al., 2021).

### 2.3 Measurement of physiological parameters

Chlorophyll a and chlorophyll b contents were determined using UV spectrophotometry (Lichtenthaler and Wellburn, 1983). Soluble sugar content was determined using the anthrone colorimetric method (Wu and Shen, 2021). Soluble protein content was determined using the Coomassie Brilliant Blue method (Snyder and Desborough, 1978). Proline content was determined using spectrophotometry (Abraham et al., 2010). Malondialdehyde (MDA) content was determined using the thiobarbituric acid (TBA) reaction method (Ding et al., 2023). Catalase (CAT) activity was determined using UV absorption spectroscopy (Dudziak et al., 2019). The activity of superoxide dismutase (SOD) was determined using the nitroblue tetrazolium (NBT) method (Giannopolitis and Ries, 1977). The activity of peroxidase (POD) was determined using the guaiacol method (Jamshidi Goharrizi et al., 2020).

### 2.4 Determination of plant hormone content

The analysis was performed using an ultra-high performance liquid chromatography coupled with a high-resolution Orbitrap mass spectrometry system (UHPLC-QE, Thermo, USA). Precisely weighed 100 mg of the sample was placed into a centrifuge tube, followed by the addition of 1 mL of pre-chilled 50% acetonitrile aqueous solution. The mixture was subjected to ultrasonic treatment at 4 °C for 3 minutes, then allowed to stand for extraction for 30 minutes. The extract was centrifuged at 12,000 rpm for 10 minutes at 4 °C, and the supernatant was collected for solid-phase extraction purification. The solid-phase extraction was conducted using a reversed-phase SPE column (RP-SPE), which was activated sequentially with 1 mL methanol and 1 mL deionized water, then equilibrated with a 50% acetonitrile aqueous solution. After loading the supernatant onto the SPE column, it was washed with 1 mL of 30% acetonitrile aqueous solution, and the eluate was collected. The collected eluate was dried under nitrogen flow to remove solvents, and the residue was resolubilized in 0.2 mL of 30% acetonitrile aqueous solution, then transferred to an autosampler vial with an inserted tube for UHPLC-QE analysis (Albaseer et al., 2010; Balcke et al., 2012; Wang et al., 2017).

### 2.5 Transcriptome analysis

Total RNA was extracted from *T. kirilowii* root tissues using TRIzol^®^ reagent (Invitrogen, USA), strictly following the standard operating procedures provided by the manufacturer (Zheng et al., 2026). RNA integrity was assessed using an Agilent 5300 Bioanalyzer (Agilent Technologies, USA), with all samples showing RNA Integrity Number (RIN) values greater than 7.0. RNA concentration and purity were determined using a NanoDrop ND-2000 spectrophotometer (Thermo Fisher Scientific, USA) (Wang et al., 2022). RNA purification, reverse transcription, cDNA library construction, and high-throughput sequencing were outsourced to Shanghai Majorbio Biotechnology Co., Ltd. (Shanghai, China), with experimental steps performed according to the respective kit manuals. Sequencing was conducted on an Illumina NovaSeq X Plus platform using paired-end (PE) 150 sequencing mode combined with the NovaSeq Sequencing Kit for data acquisition (Tian et al., 2025). Raw paired-end reads underwent quality trimming and filtering using fastp software (Chen et al., 2018). Gene expression levels were quantified using TPM (Transcripts Per Million) values. Differentially expressed genes (DEGs) were identified using the DESeq2 method, with a threshold set at |log2(FoldChange)| ≥ 1 and FDR < 0.05 (Li et al., 2026). Functional annotation and enrichment analyses of Gene Ontology (GO) terms and Kyoto Encyclopedia of Genes and Genomes (KEGG) pathways were performed using ClusterProfiler v4.0 software, with an adjusted p-value < 0.05 as the significance criterion. Enrichment results were visualized using bubble plots and clustering heatmaps (Wu et al., 2006; Yu et al., 2012).

### 2.6 Untargeted metabolomics analysis

Approximately 50 ± 5 mg of each sample was accurately weighed into a 2 mL centrifuge tube containing a 6 mm diameter grinding bead. Subsequently, 400 µL of prechilled extraction solvent (methanol-water, 4:1, v/v) was added for metabolite extraction. The sample solutions were subjected to cryogenic grinding in a freeze-tissue grinder at -10 °C with a frequency of 50 Hz for 6 minutes. Following grinding, low-temperature ultrasonic extraction was performed at 5 °C and 40 kHz for 30 minutes. After extraction completion, samples were incubated at -20 °C for 30 minutes, then centrifuged at 13,000 × g for 15 minutes at 4 °C. The supernatants were carefully transferred into vials equipped with insert tubes for instrumental analysis (Eloh et al., 2016; Wang et al., 2024). Detection and analysis were performed using a UHPLC-Exploris 240 (Thermo Fisher) system. The experiments were completed by Shanghai Majorbio Bio-pharm Technology Co., Ltd. (Shanghai, China).

### 2.7 Validation of DEGs by RT-qPCR

To validate the accuracy of the RNA-seq results, 9 DEGs were randomly selected for qRT-PCR validation. All primers are listed in **Supplementary Table 1**. cDNA synthesis was performed using a reverse transcription kit (Novazene, China), and amplification was performed using a SYBR qPCR kit (Novazene, China) on a QuantStudio 5 Real-Time PCR System (Thermo Fisher Scientific, USA). The expression levels were calculated using the 2^−ΔΔCT^ method with *T. kirilowii* 18S rRNA as the internal reference gene (Yang et al., 2023; Zheng et al., 2026).

### 2.8 Data analysis

Data are presented as mean ± standard deviation. Three biological replicates were conducted, with each replicate consisting of three plants. Data were organized in Excel 2016. Analysis of variance (ANOVA) was performed using SPSS 19.0 software (Chicago, USA).

## 3 Results

### 3.1 Morphological and physiological changes of *T. kirilowii* in response to RKN infection

**Figure 1:**
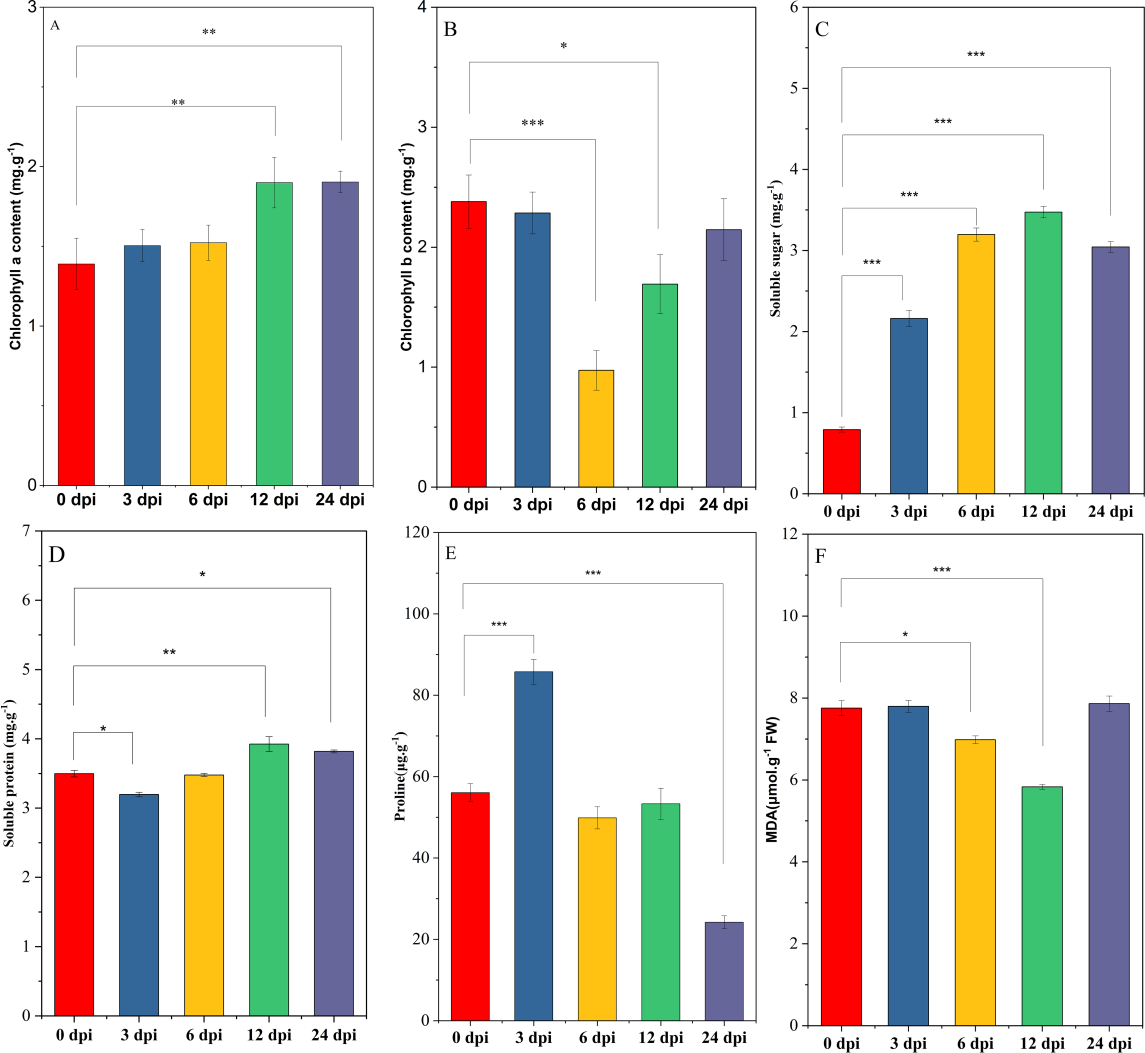
Physiological parameter changes of *T. kirilowii* infected by RKN. **(A)** Chlorophyll a content. **(B)** Chlorophyll b content. **(C)** Soluble sugar content. **(D)** Soluble protein content. **(E)** Proline content. **(F)** MDA content. *represents *P* < 0.05 **represents *P* < 0.01 ***represents *P* < 0.001.

Root-knot nematode infection affected the root growth of *T. kirilowii*. As the duration of infection increased, root galls gradually formed. At 3 dpi, no root galls were observed; by 6 dpi, root gall formation began with an average of 14 galls per plant. Thereafter, the number of galls increased significantly, reaching 50 and 69 galls per plant at 12 dpi and 24 dpi, respectively (**Supplementary Table 2**). Following RKN infection, chlorophyll a content showed a continuous upward trend, increasing by 36.7% and 37.0% at 12 dpi and 24 dpi compared to controls, respectively. In contrast, chlorophyll b content initially decreased before rising, reaching its lowest level at 6 dpi with a 59.1% reduction compared to controls (**Figures 1A, B**). These results suggest that 6 dpi may represent a critical time point for RKN infection of *T. kirilowii*. The contents of soluble sugar, soluble protein, proline, and MDA in *T. kirilowii* roots were measured at 0, 3, 6, 12, and 24 dpi. Compared to controls, soluble sugar content increased significantly at all time points after infection, with elevations of 173.2%, 303.9%, 338.9%, and 284.7%, respectively (**Figure 1C**). Soluble protein content decreased at 3 dpi, falling 8.6% below the control, then peaked at 12 dpi with a 12.2% increase compared to controls (**Figure 1D**). Proline content rose significantly at 3 dpi, increasing by 53.1% compared to the control, but declined to its lowest level at 12 dpi, decreasing by 56.8% compared to the control (**Figure 1E**). MDA content exhibited an initial decrease followed by an increase, with reductions of 9.9% and 24.8% at 6 dpi and 12 dpi compared to the control, respectively (**Figure 1F**). These findings demonstrate that RKN infection significantly alters the physiological and biochemical responses of *T. kirilowii*.

### 3.2 Antioxidant enzyme activities changes of *T. kirilowii* root in response to RKN infection

**Figure 2:**
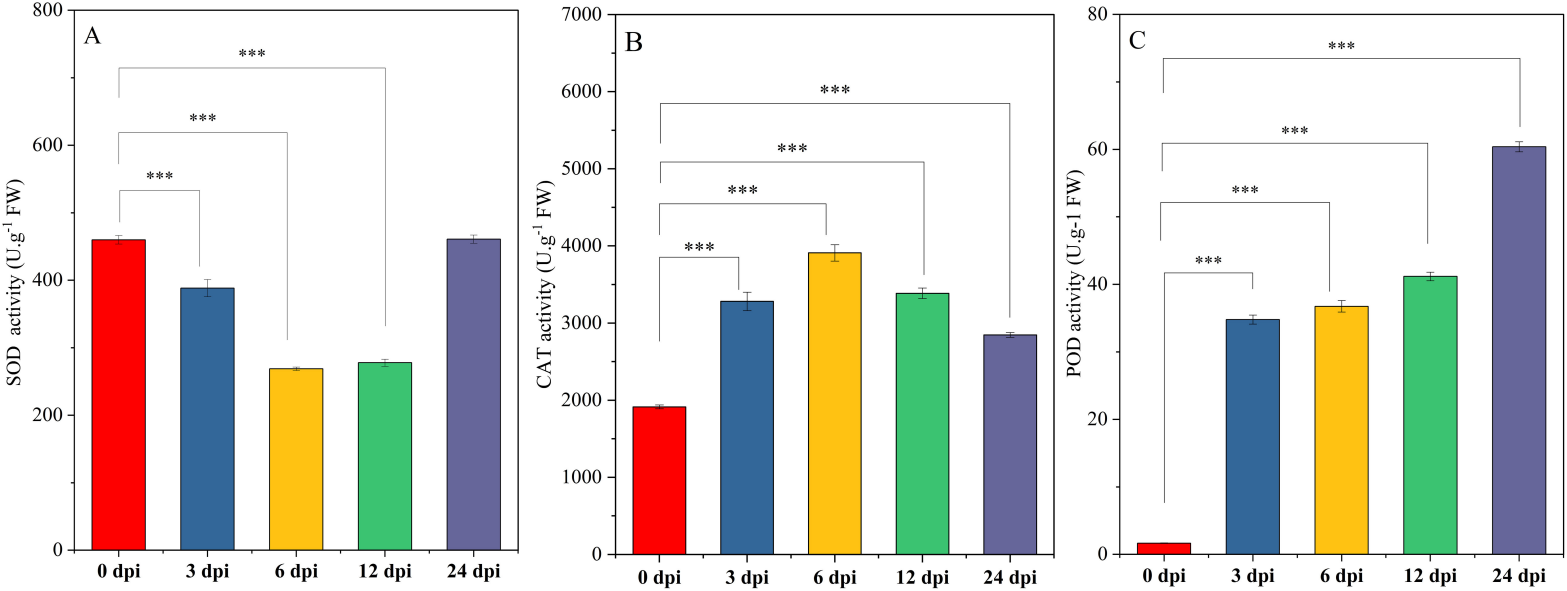
Antioxidant enzyme activity changes of *T. kirilowii* root infected by RKN. **(A)** SOD activity. **(B)** CAT activity. **(C)** POD activity. ***represents *P* < 0.001.

RKN infection significantly affected antioxidant enzyme activities of *T. kirilowii* roots (**Figure 2**). As the duration of infection increased, the activity of SOD and CAT exhibited opposite trends. SOD activity initially decreased and then increased after infection, reaching a minimum at 6 dpi, which was 41.5% lower than the control; in contrast, CAT activity peaked at 6 dpi, showing a significant increase of 104.3% compared to the control (**Figures 2A, B**). Meanwhile, POD activity was significantly higher than that of the control at all time points after infection, increasing to 21.1-fold, 22.3-fold, 24.9-fold, and 36.6-fold that of the control, respectively (**Figure 2C**).

### 3.3 Hormonal changes of *T. kirilowii* root in response to RKN infection

**Figure 3:**
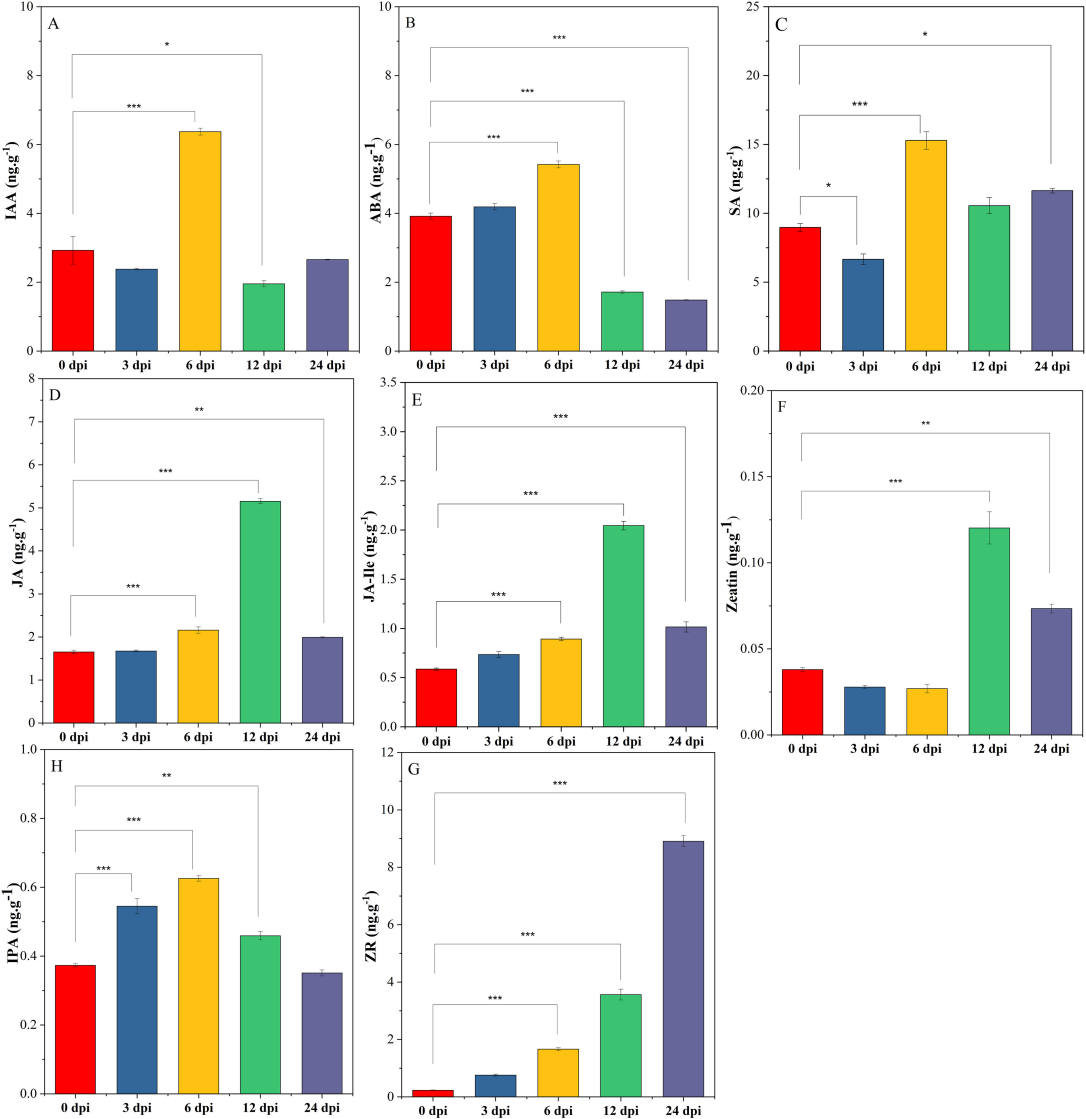
Hormone content changes of *T. kirilowii* root infected by RKN. **(A)** Indole-3-acetic acid (IAA) content. **(B)** Abscisic acid (ABA) content. **(C)** Salicylic acid (SA) content. **(D)** Jasmonic acid (JA) content. **(E)** Jasmonoyl-L-isoleucine (JA-Ile) content. **(F)** Zeatin content. **(G)** Zeatin riboside (ZR) content. **(H)** Isopentenyladenosine (IPA) content. *represents *P* < 0.05 **represents *P* < 0.01 ***represents *P* < 0.001.

To investigate the effects of RKN infection on hormone levels in *T. kirilowii* root, the contents of eight key hormones were measured at 0, 3, 6, 12, and 24 dpi. The results showed that the hormonal responses were highly time-specific (**Figure 3**). Indole-3-acetic acid (IAA) content peaked at 6 dpi with a significant increase of 117.8% compared to the control (**Figure 3A**). Abscisic acid (ABA) content exhibited an initial increase followed by a decrease, reaching its maximum at 6 dpi with a 38.4% increase compared to the control, followed by significant decreases of 56.2% and 62.1% at 12 dpi and 24 dpi, respectively (**Figure 3B**). Salicylic acid (SA) content showed an opposite pattern with an initial decrease followed by an increase, peaking at 6 dpi with a 70.4% increase compared to the control (**Figure 3C**). Jasmonic acid (JA) and its bioactive form, jasmonoyl-L-isoleucine (JA-Ile), displayed similar trends of initial increase followed by decrease, both reaching maximum levels at 12 dpi with increases of 212.3% and 248.0%, respectively, compared to control (**Figures 3D, E**). Zeatin content increased significantly at 12 dpi, with a 202.0% increase compared to the control (**Figure 3F**). Zeatin riboside (ZR) content continued to rise significantly starting at 6 dpi, reaching 38.5-fold that of the control at 24 dpi (**Figure 3G**). Isoprenyl adenosine (IPA) content showed an initial increase followed by a decline, peaking at 6 dpi with a 67.5% increase compared to the control (**Figure 3H**).

### 3.4 Transcriptome analysis of *T. kirilowii* root in response to RKN infection

**Figure 4:**
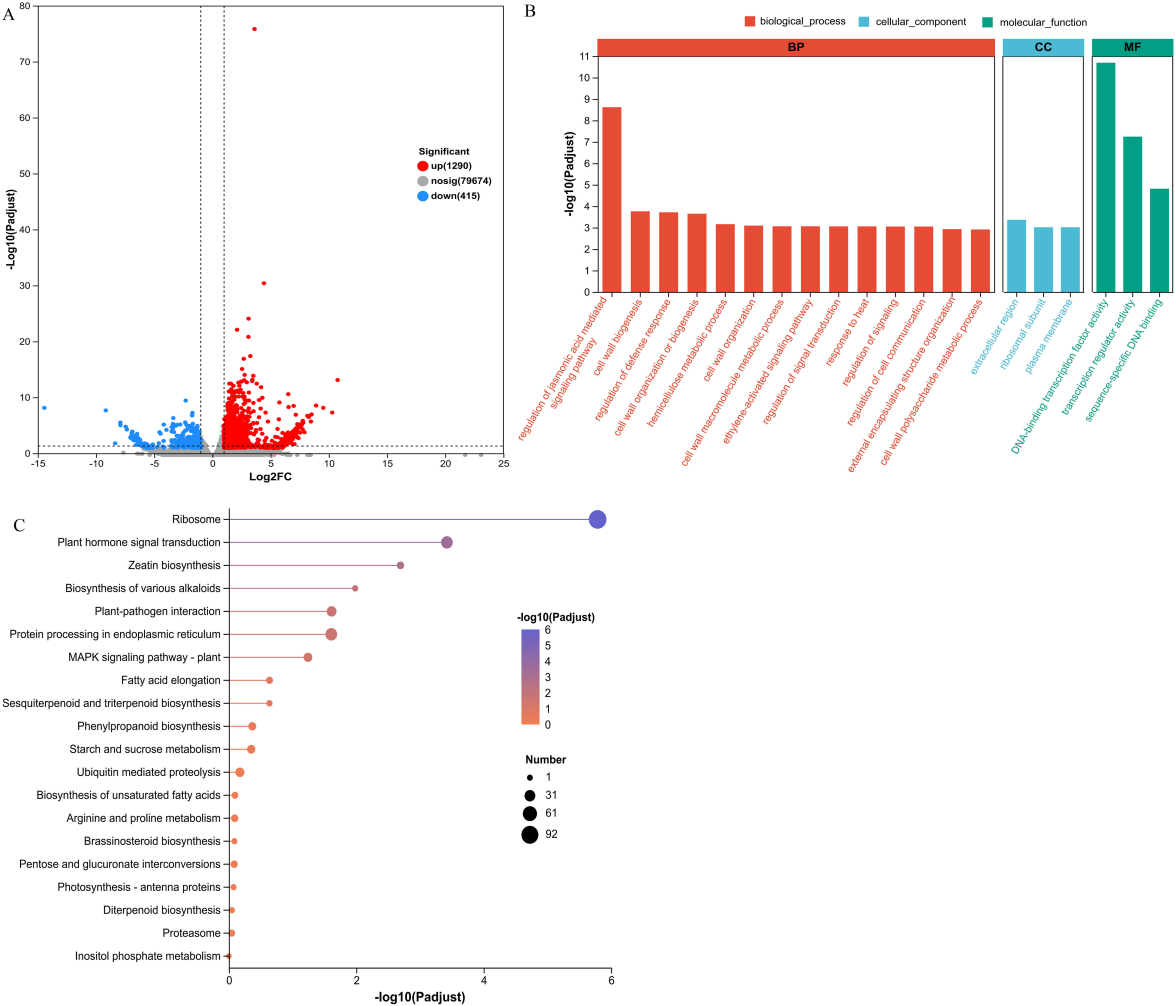
Transcriptomic analysis of *T. kirilowii* root. **(A)** Volcano plot of DEGs (6 dpi_vs_0 dpi), with red and green dots representing significantly upregulated and downregulated genes, respectively. **(B)** GO enrichment analysis of DEGs (6 dpi_vs_0 dpi). **(C)** KEGG enrichment analysis of DEGs (6 dpi_vs_0 dpi).

By analyzing the physiological response of *T. kirilowii* to RKN infection, it was determined that 6 dpi represented a critical time point for studying its response mechanisms. Therefore, RNA samples from *T. kirilowii* roots at 0 dpi and 6 dpi were sequenced to investigate the molecular mechanisms of this response. A total of 253,114,296 bp clean reads were obtained, with Q30 values exceeding 95.6% and GC content ranging from 43.98% to 44.28% (**Supplementary Table 3**), indicating reliable sequencing quality. Based on the screening criteria (|log_2_(fold change)| ≥ 1, FDR ≤ 0.01), 1,705 DEGs were identified, including 1,290 upregulated genes and 415 downregulated genes (**Figure 4A**). To investigate the potential biological functions of these DEGs, GO enrichment analysis was performed. Results showed that in biological processes (BP), DEGs were primarily enriched in the regulation of jasmonic acid mediated signaling pathway and cell wall biogenesis; in cellular components (CC), DEGs were mainly enriched in the extracellular region; and in molecular functions (MF), DEGs were significantly enriched in DNA-binding transcription factor activity, transcription regulator activity, and sequence-specific DNA binding (**Figure 4B**). To clarify the major metabolic pathways involved, KEGG enrichment analysis was conducted. The results demonstrated that DEGs were significantly enriched in key pathways, including plant hormone signal transduction, zeatin biosynthesis, alkaloid biosynthesis, and plant-pathogen interaction (**Figure 4C**).

### 3.5 Untargeted metabolomic analysis of *T. kirilowii* root in response to RKN infection

**Figure 5:**
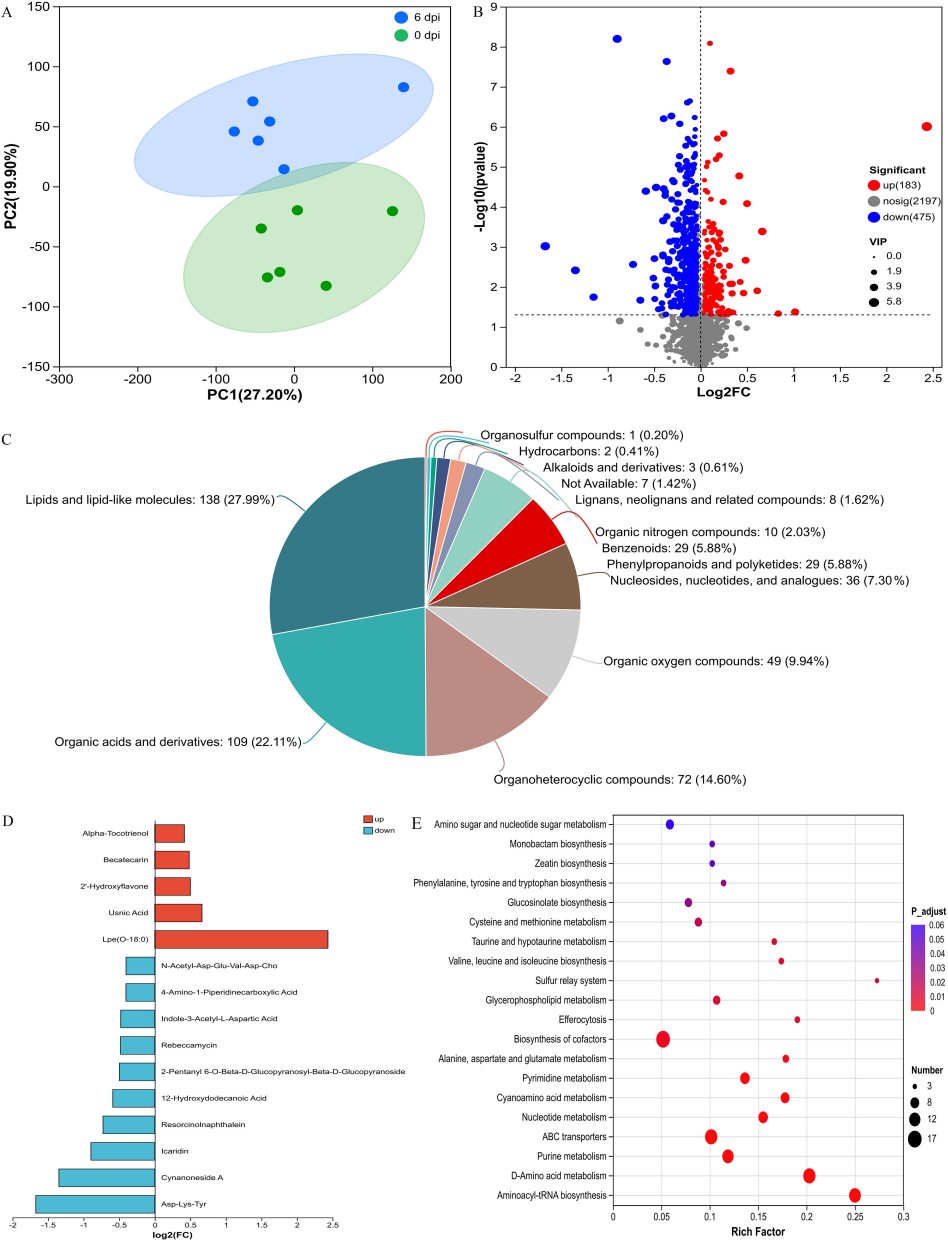
Untargeted metabolomic analysis of *T. kirilowii* root. **(A)** Principal component analysis. **(B)** Volcano plot of DAMs (6 dpi_vs_0 dpi). red and green dots indicating significantly upregulated and downregulated metabolites, respectively. **(C)** Classification analysis of DAMs (6 dpi_vs_0 dpi). **(D)** Fold change analysis of the top 15 DAMs (6 dpi_vs_0 dpi). **(E)** KEGG enrichment analysis of DAMs (6 dpi_vs_0 dpi).

To investigate the effects of RKN infection on metabolites in *T. kirilowii* roots, this study conducted untargeted metabolomics analysis. Principal component analysis (PCA) revealed a clear separation between samples from the RKN infection group and the control group (**Figure 5A**). Based on screening criteria for differentially accumulated metabolites (DAMs) (VIP > 1 and |log_2_(fold change)| ≥ 1), a total of 658 DAMs were identified, including 475 upregulated and 183 downregulated metabolites (**Figure 5B**). Classification analysis showed these DAMs primarily belonged to carboxylic acids and derivatives, organic oxygen compounds, amino acids, phospholipids, fatty acid esters, and isoprenoid alcohol esters (**Figure 5C**). Among the top 15 metabolites ranked by significance of fold changes, 5 were significantly upregulated and 10 were significantly downregulated. The significantly upregulated metabolites included Lpe (O-18:0), Usnic Acid, 2’-Hydroxyflavone, Becatecarin, and Alpha-Tocotrienol, while the significantly downregulated metabolites comprised Asp-Lys-Tyr, Cynanoneside A, Icaridin, Resorcinolnaphthalein, 12-Hydroxydodecanoic Acid, Pentanyl 6-O-Beta-D-Glucopyranosyl-Beta-D-Glucopyranoside, Rebeccamycin, Indol-3-Acetyl-L-Aspartic Acid, 4-Amino-1-Piperidinecarboxylic Acid, and N-Acetyl-Asp-Glu-Val-Asp-Cho (**Figure 5D**). To identify key metabolic pathways enriched by these DAMs, KEGG enrichment analysis was performed. Results demonstrated significant enrichment in 19 pathways, including phenylalanine, tyrosine, and tryptophan biosynthesis; cysteine and methionine metabolism; valine, leucine, and isoleucine biosynthesis; zeatin biosynthesis; and ABC transporters (**Figure 5E**).

### 3.6 Integrated transcriptomic and untargeted metabolomic analysis of *T. kirilowii* root in response to RKN infection

**Figure 6:**
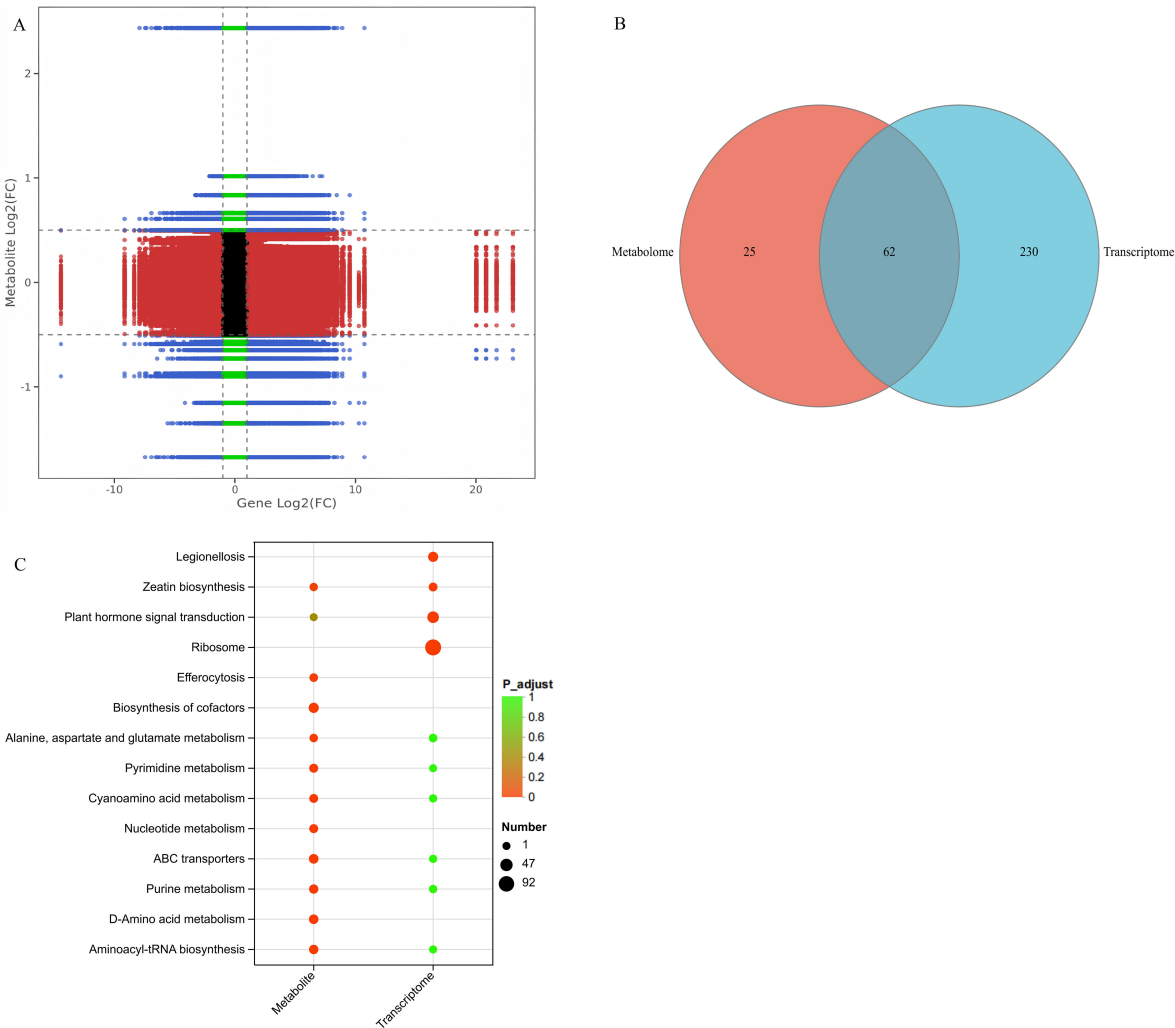
Integrated analysis of transcriptome and untargeted metabolome. **(A)** Nine-quadrant plot of transcriptome and untargeted metabolome. Red indicates upregulation of both genes and metabolites; green represents downregulation of both genes and metabolites; black indicates statistically insignificant associations; and blue represents opposite expression trends betweengenes and metabolites. **(B)** Venn diagram of transcriptome and untargeted metabolome. **(C)** KEGG enrichment analysis of transcriptome and untargeted metabolome (6 dpi_vs_0 dpi).

To systematically elucidate the response mechanisms of *T. kirilowii* root in response to RKN infection, an integrated transcriptomic and metabolomic analysis was performed. DEGs and DAMs exhibited significant correlation patterns in a nine-quadrant plot, with consistent changes in transcriptomic and metabolomic profiles observed in the third and seventh quadrants (**Figure 6A**). Venn diagram analysis revealed that DEGs and DAMs shared 62 co-enriched pathways (**Figure 6B**). Further KEGG co-enrichment analysis indicated that the zeatin biosynthesis pathway was the only significantly co-enriched pathway (**Figure 6C**).

#### 3.6.1 Zeatin biosynthesis pathway analysis

**Figure 7:**
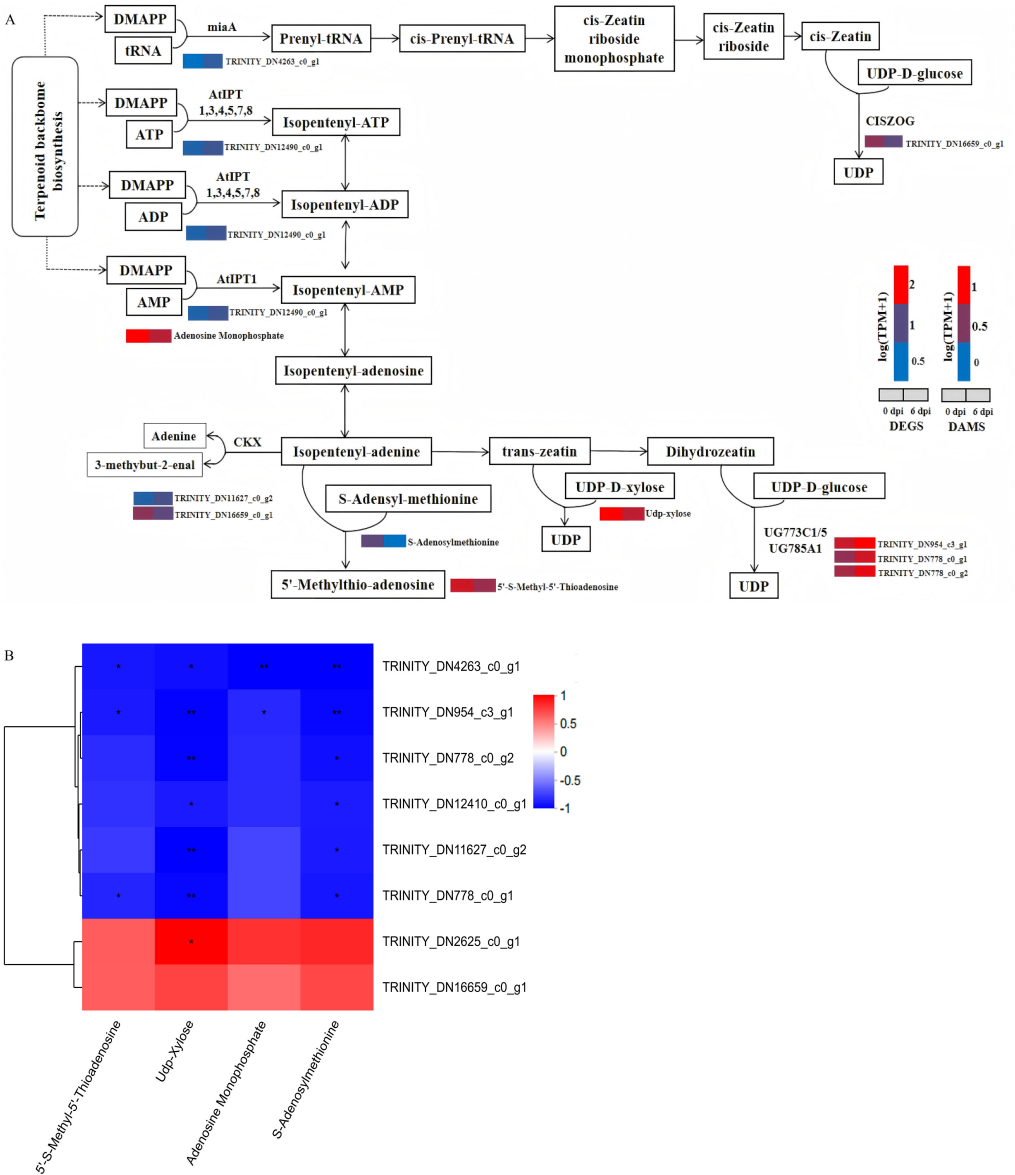
Effects of RKN infection on zeatin biosynthesis. **(A)** Pathway diagram of zeatin biosynthesis. Red indicates upregulation of metabolites or genes; blue represents downregulation of metabolites or genes. **(B)** Expression correlation heatmap.*represents *P* < 0.05 **represents *P* < 0.01.

RKN infection significantly reprograms the zeatin biosynthesis pathway, involving significant changes in 8 related genes and 5 metabolites. In the trans-zeatin synthesis pathway, isoprenyltransferase gene expression was significantly upregulated, accompanied by decreased adenosine monophosphate (AMP), suggesting a limited precursor supply of isoprenylated adenosine. Meanwhile, downstream UDP-glycosyltransferase gene expression showed significant upregulation, driving substantial accumulation of dihydrozeatin O-glucoside, indicating that infection activated glycosylation modification and storage processes of trans-zeatin. In the cis-zeatin branch, tRNA isoprenyltransferase gene expression was significantly upregulated, while the key inactivation enzyme *CISZOG* for cis-zeatin demonstrated significant downregulation, synergistically promoting accumulation of active cis-zeatin and its nucleoside forms. This coordinated upregulation of synthesis coupled with downregulation of degradation creates a potent regulatory module that promotes cis-zeatin accumulation. Additionally, substrate UDP-D-xylose levels in the zeatin xylosylation pathway were significantly reduced. Methionine cycle-related metabolites exhibited complex regulatory patterns, with both S-adenosylmethionine (SAM) and 5’-methylthioadenosine levels significantly downregulated; adenine metabolism-related enzymes displayed mixed regulation modes; and 3-methyl-2-butenal metabolism showed coexisting upregulation and downregulation, reflecting metabolic reprogramming of methyl donor cycles under infection (**Figure 7A**). Collectively, these coordinated changes in zeatin biosynthesis pathway genes and metabolites are functionally coherent, driving net cis-zeatin accumulation to stimulate cell cycle progression and meristematic activity within giant cells and surrounding gall tissues, thereby providing a mechanistic link between metabolic reprogramming and nematode feeding site formation. Correlation analysis between differential metabolites and genes revealed that 5’-S-Methyl-5’-thioadenosine was significantly negatively correlated with TRINITY_DN4263_c0_g1, TRINITY_DN954_c3_g1, and TRINITY_DN778_c0_g2; UDP-Xylose showed significant negative correlations with TRINITY_DN4263_c0_g1 and TRINITY_DN12410_c0_g1, highly significant negative correlations with TRINITY_DN954_c3_g1, TRINITY_DN11627_c0_g2, TRINITY_DN778_c0_g1, and TRINITY_DN778_c0_g2, and a significant positive correlation with TRINITY_DN2625_c0_g1; Adenosine Monophosphate exhibited a significant negative correlation with TRINITY_DN4263_c0_g1 and a highly significant negative correlation with TRINITY_DN954_c3_g1; S-Adenosylmethionine demonstrated significant negative correlations with TRINITY_DN12410_c0_g1, TRINITY_DN11627_c0_g2, TRINITY_DN954_c3_g1, TRINITY_DN778_c0_g1, and TRINITY_DN778_c0_g2 and showed highly significant negative correlations with TRINITY_DN4263_c0_g1 and TRINITY_DN954_c3_g1 (**Figure 7B**).

#### 3.6.2 qRT-PCR validation of DEGS

**Figure 8:**
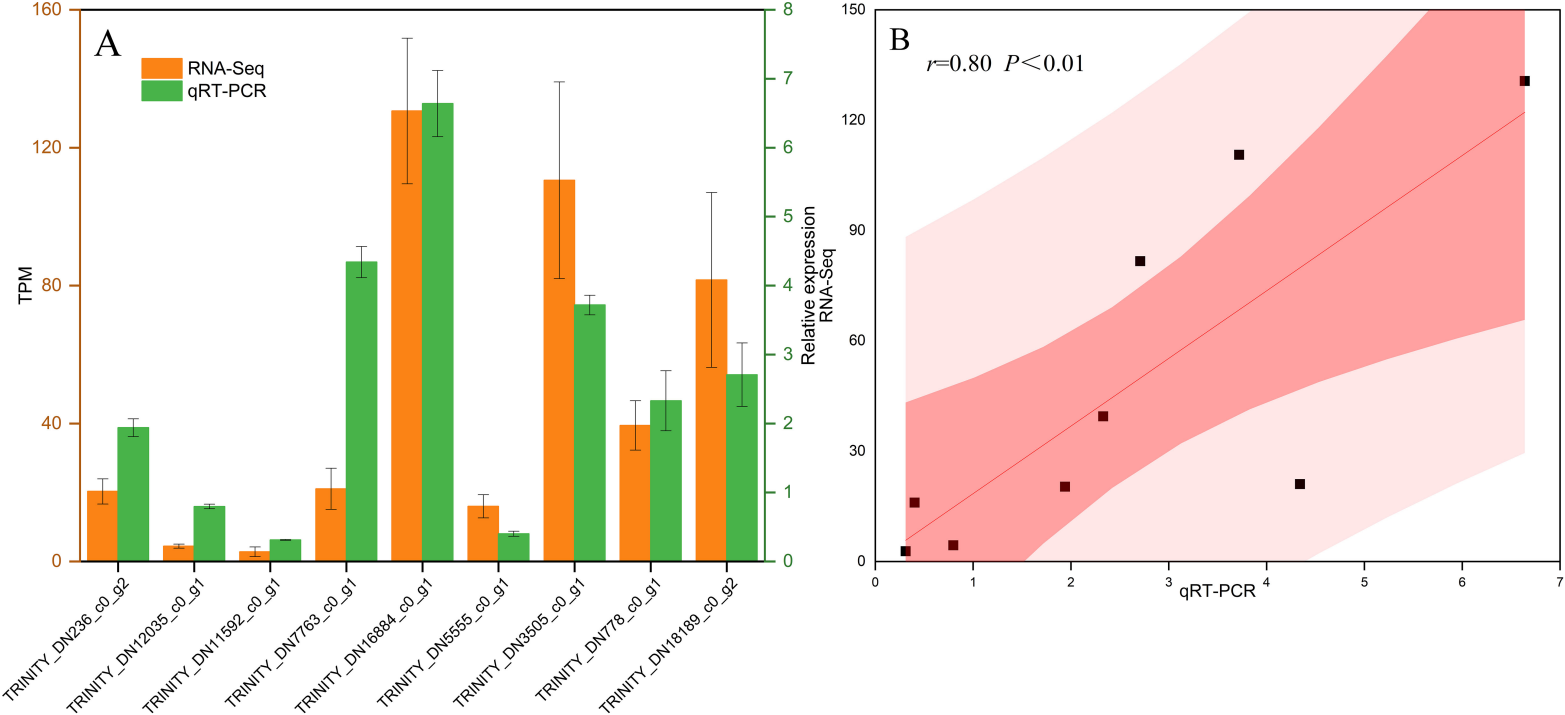
Comparison of RNA-seq and qRT-PCR results **(A)** Bar chart of RNA-seq and qRT-PCR results. **(B)** Correlation analysis of RNA-seq and qRT-PCR results.

To validate the reliability of the RNA-seq data, nine significantly differentially expressed genes were randomly selected from the DEGs identified in *T. kirilowii* roots infected by RKN at 6 dpi. The expression levels of these genes were analyzed using qRT-PCR. The results demonstrated that the expression patterns of all nine DEGs were consistent with the RNA-Seq data, confirming the high reliability of the transcriptome sequencing data (**Figure 8A**). Correlation analysis further revealed a strong positive correlation between the transcriptome sequencing data and qRT-PCR results (r = 0.80, *P* < 0.01) (**Figure 8B**).

### 3.7 Correlation analysis of key genes and core metabolites with *T. kirilowii* morphological and physiological traits

**Figure 9:**
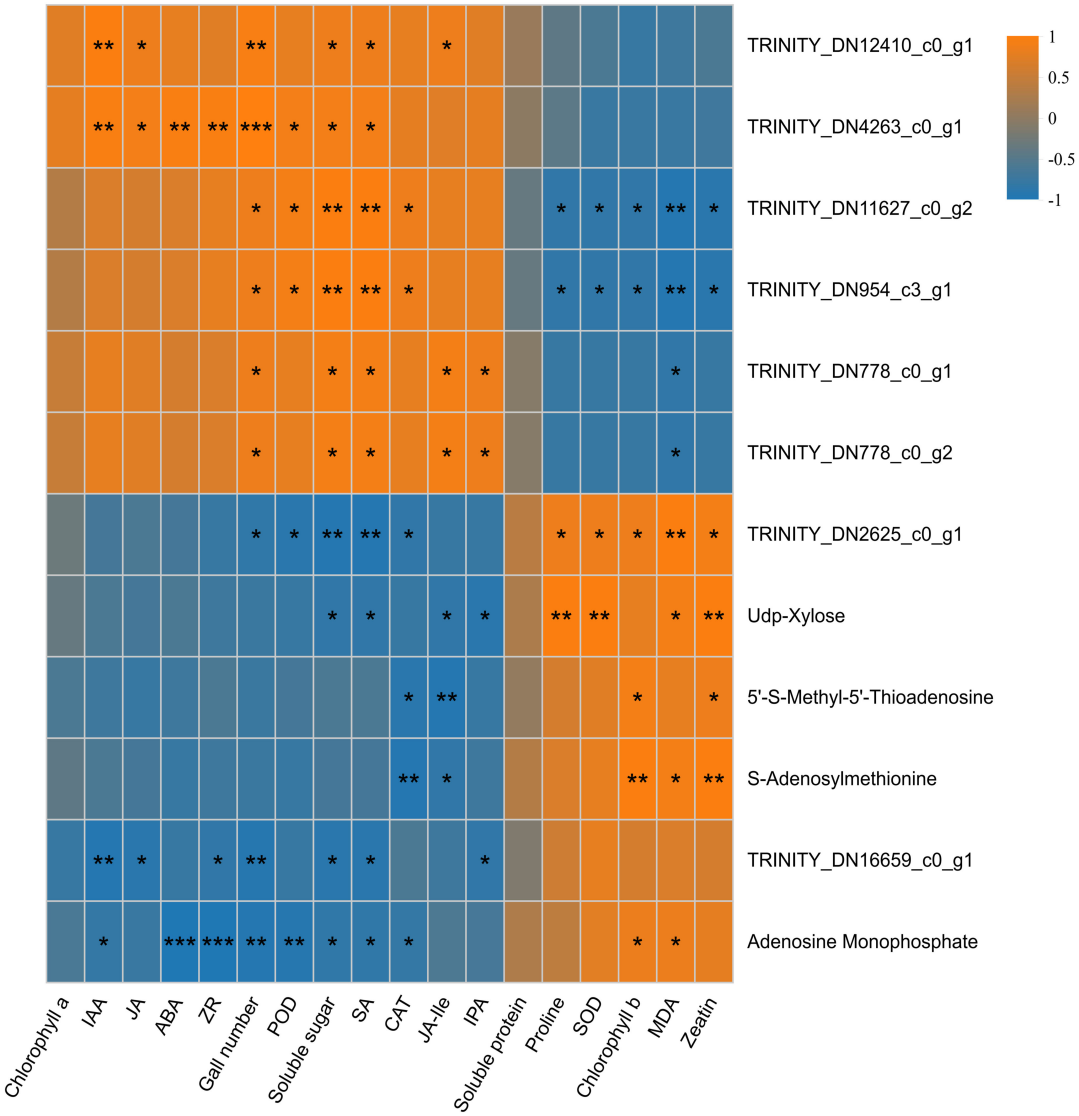
Correlation analysis among key genes, core metabolites, and morphological and physiological traits. *represents *P* < 0.05 **represents *P* < 0.01 ***represents *P* < 0.001.

A heatmap based on Pearson correlation analysis was generated to illustrate the relationships among eight key genes and four core metabolites in the zeatin biosynthesis pathway and morphological traits, physiological parameters, and hormone contents (**Figure 9**). At the key gene level, TRINITY_DN12410_c0_g1 exhibited significant positive correlations with IAA, JA, Gall number, soluble sugar, SA, and JA-Ile; TRINITY_DN4263_c0_g1 showed significant positive correlations with IAA, JA, ABA, ZR, Gall number, POD, soluble sugar, and SA; TRINITY_DN11627_c0_g2 and TRINITY_DN954_c3_g1 demonstrated significant positive correlations with Gall number, POD, soluble sugar, SA, and CAT, while showing significant negative correlations with proline, SOD, chlorophyll b, MDA, and zeatin; however, TRINITY_DN2625_c0_g1 exhibited a completely opposite correlation pattern; TRINITY_DN778_c0_g1 and TRINITY_DN778_c0_g2 were significantly positively correlated with Gall number, soluble sugar, SA, JA-Ile, and IPA, and negatively correlated with MDA; TRINITY_DN16659_c0_g1 showed significant negative correlations with IAA, JA, ZR, Gall number, soluble sugar, SA, and IPA. At the core metabolite level, UDP-xylose was significantly negatively correlated with soluble sugar, SA, JA-Ile, and IPA and significantly positively correlated with proline, SOD, MDA, and zeatin; 5’-S-Methyl-5’-thioadenosine was significantly negatively correlated with CAT and JA-Ile and significantly positively correlated with chlorophyll b and zeatin; S-Adenosylmethionine demonstrated significant negative correlations with CAT and JA-Ile and was significantly positively correlated with chlorophyll b and zeatin; adenosine monophosphate exhibited significant negative correlations with IAA, ABA, ZR, gall number, POD, soluble sugar, SA, and CAT, while showing significant positive correlations with chlorophyll b and MDA.

## 4 Discussion

### 4.1 Morphological and physiological changes of *T. kirilowii* in response to RKN infection

RKN infection significantly disrupted the morphological and physiological homeostasis of the host plant *T. kirilowii* root. This study demonstrated that galls appeared at 6 dpi, followed by a significant increase in gall numbers. This pattern is similar to the infection process of RKNs in crops such as tomato and soybean, but the rate of gall formation is relatively slower, reflecting significant differences in host susceptibility to RKNs (Collett et al., 2023; Xu et al., 2019). Chlorophyll content changes reveal photosynthetic adaptation processes in plants. After RKN infection, chlorophyll a content in *T. kirilowii* leaves continuously increased, while chlorophyll b content initially decreased and then increased. This pattern differs from the results observed in muskmelon and ridge gourd, which may be related to the sampling time intervals (Basavaraj et al., 2025; Pandey and Nayak, 2018). The results of this study indicate that RKN infection initially caused short-term suppression of photosynthetic function in *T. kirilowii*, but the host subsequently activated compensatory mechanisms to maintain energy supply. Soluble sugar content continued to rise significantly throughout the infection period, consistent with reports on tobacco and tomato following RKN infection (Sun et al., 2024; Yang et al., 2025), suggesting that sugar metabolism reprogramming represents a conserved strategy for plants to cope with RKN infection. Notably, unlike the exacerbation of oxidative damage commonly reported in other studies, MDA content in *T. kirilowii* root significantly decreased at 6 dpi and 12 dpi, while CAT activity peaked at 6 dpi and POD activity continued to increase substantially (Danish et al., 2021; Khanna et al., 2019; Korayem et al., 2012). This antioxidant response pattern indicates that *T. kirilowii* may effectively alleviate oxidative stress induced by RKN infection through the efficient activation of a non-enzymatic antioxidant defense system dominated by CAT and POD, which may be related to its long-term evolutionary adaptation as a medicinal plant. The multiple significant morphological and physiological changes observed at 6 dpi indicate that this time point represents a critical response node for RKN infection of *T. kirilowii* root.

### 4.2 Hormone signal regulation of *T. kirilowii* root in response to RKN infection

RKN infection triggered significant hormonal changes in *T. kirilowii* roots with pronounced temporal specificity. JA and its bioactive conjugate JA-Ile peaked at 12 dpi, coinciding with the significant enrichment of JA-mediated signaling pathways in the transcriptome. This observation is consistent with findings from rice and tomato studies identifying JA as a key positive regulator of root-knot nematode resistance (Fan et al., 2014; Lahari et al., 2019). However, deviating from the classical SA-JA antagonism model, SA content also increased significantly at 6 dpi, suggesting potential synergistic rather than purely antagonistic interactions between SA and JA signaling. This pattern is consistent with evidence that SA-JA crosstalk is context-dependent, where NPR1-mediated SA signaling can cooperate with JA pathways through shared transcriptional targets under specific biotic stresses (Li et al., 2019). This difference in hormonal interaction patterns may reflect the differentiation of response strategies among different host plants to RKN infection. Furthermore, the significant peak of IAA content at 6 dpi closely coincides with the onset of gall formation, supporting the critical role of IAA in giant cell formation and gall development (Ali et al., 2025; Noureddine et al., 2023; Wang et al., 2018). Among the 1,290 upregulated genes at 6 dpi, core auxin-responsive DEGs (GH3.17, SAUR50, and AUX/IAA) and the SCF-TIR1 component ASK10 were identified, indicating transcriptional engagement of auxin perception and signaling during gall initiation. The dynamic pattern of ABA, featuring an initial increase followed by a decline, may reflect the activation of defense responses such as stomatal closure during early infection stages, coupled with adaptive adjustments to water transport impairment caused by later gall formation. Notably, cytokinin hormones exhibited exceptionally high accumulation in infected roots. Zeatin content increased by 202% at 12 dpi, while ZR reached 38.5-fold that of the control by 24 dpi. Studies have shown that cytokinins play a key regulatory role in plant-RKN interactions by promoting cell division and differentiation in gall tissues and maintaining cell viability at the infection site (Dowd et al., 2017; Olmo et al., 2020). Compared to previously reported host plants, the magnitude of ZR accumulation in *T. kirilowii* roots was particularly pronounced, which may reflect a specific hormonal response pattern of *T. kirilowii* in response to RKN infection.

### 4.3 Transcriptome-metabolome integration analysis reveals a complex regulatory response system

Transcriptomic analysis identified 1,705 DEGs primarily enriched in functional categories including jasmonic acid-mediated signaling pathway regulation, cell wall biogenesis, and DNA-binding transcription factors, reflecting multi-layered activation of defense mechanisms. Untargeted metabolomics identified 658 DAMs mainly involving carboxylic acid derivatives, organo-oxygen compounds, and amino acids, representing the transformation process from gene expression to phenotypic output. Integrated transcriptome-metabolome analysis demonstrated that the zeatin biosynthesis pathway was the only significantly co-enriched pathway. This contrasts with previous studies focusing on jasmonic JA and SA roles in RKN interactions; this study’s findings revealed the central role of zeatin metabolism in *T. kirilowii* response to RKN infection (Du et al., 2021; Yang et al., 2018). Specifically, upregulation of isoprenyltransferase genes in the trans-zeatin synthesis pathway is accompanied by a reduced AMP level, suggesting a limitation in precursor supply. Concurrently, the upregulation of UDP-glucosyltransferase genes drives the accumulation of dihydrozeatin O-glucoside, indicating that infection activates a glycosylation-based storage mechanism for zeatin. More critically, within the cis-zeatin branch, the upregulation of tRNA isopentenyltransferase genes coupled with the downregulation of *CISZOG* creates a synergistic expression pattern that promotes the accumulation of active cis-zeatin and its nucleoside forms. These findings are consistent with recent reports on the unique regulatory role of cis-zeatin in plant stress responses, suggesting that cis-zeatin may be a key active molecule regulating the *T. kirilowii*-RKN interaction (Grosskinsky et al., 2013; Yousaf et al., 2024). The temporal coincidence of cis-zeatin accumulation with gall proliferation suggested a potential role in feeding site maintenance, though functional validation is required to distinguish its pro-susceptibility versus pro-defence activities. Significant downregulation of SAM and 5’-methylthioadenosine, metabolites associated with the methionine cycle, indicates metabolic reprogramming of the methyl donor cycle, which may affect epigenetic regulatory processes such as DNA methylation, providing new insights for a deeper understanding of the *T. kirilowii*-RKN interaction. Further investigation is needed to clarify the epigenetic consequences of SAM downregulation, including DNA methylation and histone modification dynamics at defence gene loci. Correlation analysis revealed significant associations between key genes and metabolites of the zeatin biosynthesis pathway and gall numbers, soluble sugars, MDA, and various hormones, indicating that this pathway plays a pivotal role in integrating stress signals, metabolic reprogramming, and morphogenesis. Nevertheless, bulk RNA-seq averages transcriptional signals across heterogeneous gall tissues, masking distinct responses of giant cells, cortical cells, and vascular elements to infection. Single-cell and spatial transcriptomics now resolve such compartmentalized programs at cellular resolution, providing a framework to dissect zeatin biosynthesis dynamics during RKN infection (Ali et al., 2026).

## 5 Conclusions

This study systematically elucidated the multi-level regulatory response system of *T. kirilowii* to RKN infection through multi-omics analysis. The results indicate that 6 dpi is a critical response node, and the zeatin biosynthesis pathway serves as a central hub for integrating hormonal signals and metabolic reprogramming. Unlike the classical JA/SA-centric defense model reported in most plant–RKN systems, this study identifies zeatin metabolism as the primary pathway hub in T. kirilowii against RKN infection, providing a new mechanistic paradigm for understanding medicinal plant resistance to RKNs. This research provides a foundation for molecular breeding of *T. kirilowii* resistant to RKNs and for developing control strategies based on zeatin regulation. However, the functions of key differentially expressed genes identified in the zeatin biosynthesis pathway require validation through genetic approaches, and the findings obtained under *in vitro* culture conditions need confirmation through field experiments. Future studies should integrate single-cell and spatial transcriptomics to resolve cell-type-specific zeatin pathway partitioning and dissect the transcriptional regulatory network downstream of cis-zeatin signaling and its crosstalk with other hormonal signals. In summary, this study reveals an integrated mechanism involving physiological regulation, hormonal coordination, metabolic reprogramming, and molecular defense responses in *T. kirilowii* against RKN infection, with the zeatin biosynthesis pathway playing a central role. These findings offer significant insights into the interaction mechanisms between medicinal plants and RKNs and provide a foundation for the development of novel strategies for controlling RKN disease.

## Data Availability

The datasets presented in this study can be found in online repositories. The names of the repository/repositories and accession number(s) can be found at https://www.ncbi.nlm.nih.gov/, PRJNA1395323.
